# Capillary Filling at the Microscale: Control of Fluid Front Using Geometry

**DOI:** 10.1371/journal.pone.0153559

**Published:** 2016-04-22

**Authors:** C. Trejo-Soto, E. Costa-Miracle, I. Rodriguez-Villarreal, J. Cid, T. Alarcón, Aurora Hernández-Machado

**Affiliations:** 1 Departament ECM, Facultat de Física, Universitat de Barcelona, Diagonal 645, E-08028 Barcelona, Spain; 2 Centre de Recerca Matemàtica, Edifici C, Campus de Bellaterra, 08193 Bellaterra (Barcelona), Spain; 3 Departament de Matemàtiques, Universitat Autònoma de Barcelona, 08193 Bellaterra (Barcelona), Spain; 4 Barcelona Graduate School of Mathematics (BGSMath), Barcelona, Spain; 5 Servicio de Hemoterapia y Hemostasia, Hospital Clínic de Barcelona, Barcelona, Spain; 6 ICREA (Institució Catalana de Recerca i Estudis Avana̧ts), Barcelona, Spain; Texas A&M University, UNITED STATES

## Abstract

We propose an experimental and theoretical framework for the study of capillary filling at the micro-scale. Our methodology enables us to control the fluid flow regime so that we can characterise properties of Newtonian fluids such as their viscosity. In particular, we study a viscous, non-inertial, non-Washburn regime in which the position of the fluid front increases linearly with time for the whole duration of the experiment. The operating shear-rate range of our apparatus extends over nearly two orders of magnitude. Further, we analyse the advancement of a fluid front within a microcapillary in a system of two immiscible Newtonian liquids. We observe a non-Washburn regime in which the front can accelerate or decelerate depending on the viscosity contrast between the two liquids. We then propose a theoretical model which enables us to study and explain both non-Washburn regimes. Furthermore, our theoretical model allows us to put forward ways to control the emergence of these regimes by means of geometrical parameters of the experimental set-up. Our methodology allows us to design and calibrate a micro-viscosimetre which works at constant pressure.

## Introduction

The study of fluid flow at the micro- and nano-scale [1, 2] has been the engine of a large number of new technologies, such as high-throughput techniques [3, 4], drug delivery [5], and computation, encryption and biological processing [6]. These technologies are based on the control and manipulation of objects such as droplets, cells, vesicles etc. [7–10]. Driven by the potential applications of miniaturised devices in industrial and medical instruments, control of fluid flow through microchannels is one the most currently studied topics in microfluidics. An additional advantage of the use of microdevices is related to the lower costs associated to small amount of materials needed, portability, and fast data acquisition. Specifically, in the field of microfluidics, it is well documented the use of devices as viscometers. Examples of such contributions include [11], where a microviscometer is developed where the fluid to be studied flows side by side with a reference fluid of known viscosity. Other devices reported in the literature are based on using the capillary-driven motion of a liquid front inside a microchannel for the measurement of the viscosity of the fluid [12]. Concerning other methods based on particle tracking (e.g. micro particle image velocimetry), Degre et al. [13] have addressed the study of rheological properties of complex fluids in microchannels.

Besides potential technological applications, there are basic questions that remain to be understood, in particular those related to the dynamics of the moving contact line [8, 14–17]. In this context, a classical result dates back to the work of Washburn who showed that, in the quasi-steady regime, the average advancement of the front in a capillary microtube obeys a diffusive dynamics, i.e. *h* ∼ *t*^*ν*^ where *h* is the position of the meniscus and the exponent *ν* = 1/2 [18].

**Fig 1:**
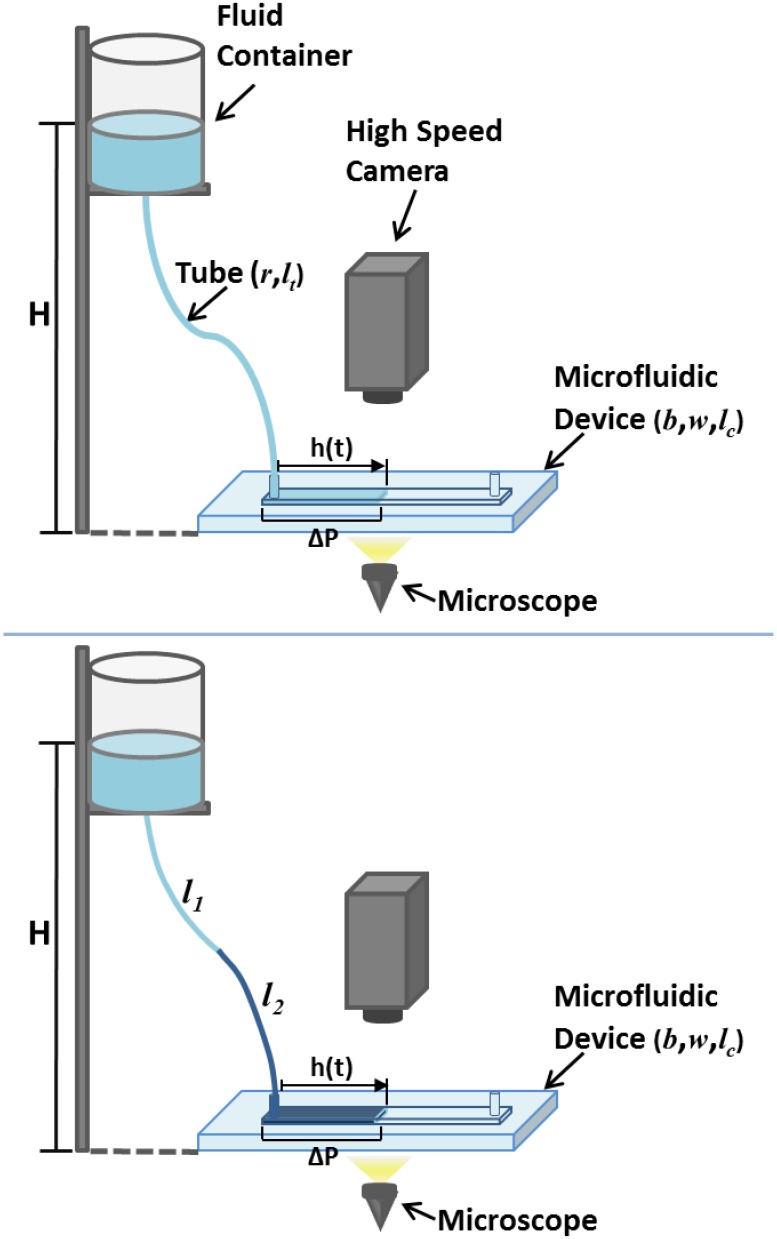
Schematic representation of the experimental set up. The upper pannel shows the single-fluid setup whereas the bottom figure shows the two-fluid setup.

**Fig 2:**
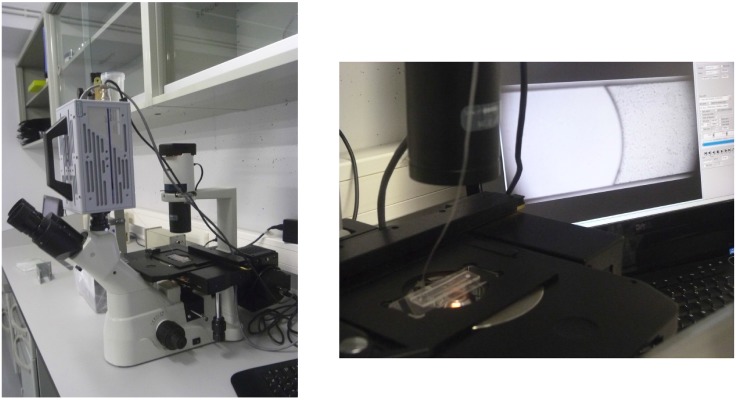
Photographs showing the actual experimental setup in our laboratory. The left pannel is a general view. The right photo shows a more detailed view of the microchannel being fed by the inlet tube.

Our aim in this paper is to propose a method of control of fluid flow in a microchannel. This enables us to analyse a flow regimes different from that studied by Washburn. Within these regimes, we show that we can characterise rheological properties, e.g. viscosity, of the fluid which is particularly suitable for potential automatisation. In a typical situation, where a single fluid is pushed through a micro-channel, we show that a set-up (see Figs 1 & 2) is possible where, after the inertial regime has decayed, whose typical time scale in microchannels is of the order of 10^−5^ s, and prior to the Washburn quasi-steady regime, a transient, viscous regime ensues such that *h* ∼ *t*, i.e. the front propagates at constant speed. We further propose a method to experimentally control the cross-over time between our transient and the Washburn regimes so that we can make the duration of the transient regime to last for an arbitrarily long time, in particular, until the microchannel is completely full.

The fact that within the transient regime the front is moving with constant velocity enables us to simplify the experimental measure of the viscosity. It can be therefore used to propose a new type micro-rheometer based on the measurement of the position of the front in contrast to methods based on tracer particles such as micro particle image velocimeters (*μ*PIVs).

In addition, we extend our framework to the analysis of a two-fluid system, where we identify a non-Washburn fluid-flow regime where the fluid front undergoes a transient acceleration. One of the advantages of using micro- and nano-technological devices, such as lab-on-a-chip experiments, is the small amount of often very expensive or difficult to come by substances. However, it is often the case that one needs this valuable liquid to fill up a whole experimental device before it enters the capillary where the actual analysis is performed. Besides costs, there are other factors to take into account. For example, in the case of blood, one may observe sedimentation before it enters the capillary of the lab-on-a-chip device where measurements are carried out. One possible way to circunvent these issues is to use two immiscible fluids in the setup shown in Fig 1. We use a cheap, fully-characterised liquid which fills the parts of the device other than the microcapillary and pushes into the capillary the fluid whose properties we wish to investigate.

This situation justifies the analysis of the dynamics of front advancenment in a system consisting of an immiscible two-fluid flow. We focus on the simplest situation in which both fluids are Newtonian and with non-zero viscosity contrast.

Our aim in this paper is to present a general framework which allows us to study both non-Washburn regimes in a unified way. We further use this framework to propose two methodologies to measure the viscosity for both the one-fluid and the two-fluid systems.

## Materials and methods

### Experimental setup

We study fluid flow inside a capillary microchannel using a pressure-driven flow. In this method, a pressure difference is set and the velocity of the fluid-air interface inside the microchannel is measured.

The microsystem is a rectangular microchannel. We consider microchannels of different heights: *b* = 300 *μ*m, *b* = 200 *μ*m, *b* = 150 *μ*m, and *b* = 50 *μ*m, and width *w* = 1 *mm* and length *l*_*c*_ = 4 *cm*, molded in a biocompatible hydrophobic silicone, PDMS (polydimethylsiloxane), on a glass substrate according to Replica Moulding and Soft Lithography microfabrication methods [19, 20]. The bottom surface of the microchannel is made of glass and the top and lateral surfaces are made of PDMS. The inlet and the outlet are perpendicular holes set at each extremity of the channel. The observation of the flow inside the microchannel is made by an inverted microscope (Optika XDS-3) with a 10× objective and a high speed camera (Photron Fastcam SA3), recording between 1000 and 125 frames per second. This gives a minimal time between 2 contiguous images of 0.001 *s* and 0.008 *s* depending of the chosen frame rate. The 10× objective allows us a 2 *mm* view of the channel. Photographs of the experimental setup are shown in Fig 2. The pressure is controlled through a fluid column inside a reservoir set at heights, H, ranging from *H* = 0.420 to 0.050 *m* and connected to a microtube of biocompatible material with uniform internal circular cross-section of diameter *d* = 0.254 *mm* and length *l*_*t*_ = 0.430 *m* (Fig 1). Note that the gap of the microchannel and the diameter of the microtube are comparable. The velocity of the fluid is obtained by tracking the mean fluid front position as a function of time between several contiguous images and averaging its values thought the channel length.

We study the behavior of three fluids of different viscosities and densities: water, ethylene glycol at two different volume concentrations and blood plasma. Plasma was obtained from human blood samples from two anonymous donors randomly selected. Blood samples were delivered for our experiments from the blood bank of the Hematology Department of the Clinical Hospital of Barcelona, in tubes of 10 or 5 *ml* on an heparine based anticoagulant. The use of these samples was authorised by the Bioethics Committee of the University of Barcelona. In order to preserve the state of the samples their are stored on a refrigerator at 4*C*. Before any intervention, the sample is set under an extraction hood to acquire room temperature, between 20 to 25°C. To separate the plasma from the cellular fraction, the blood sample in the tube is set on a centrifuge and spun for 5 minutes at 2500 *rpm*. When the spinning has finished, the cellular fraction (RBC’s, WBC’s and platelets) is confined at the bottom of the tube and the plasma on the top. The plasma is extracted using an sterile pipette, under the extraction hood to avoid contamination of the sample.

In a second set of experiments, we consider a two-fluid system where we fill the reservoir with a liquid of known viscosity, *η*_1_, (a glycerol solution at 19% of volume concentration). We then study the properties of the dynamics of the fluid front of a second fluid of unknown viscosity, *η*_2_, which is being pushed into the microchannel by a column of glycerol solution. We have used two Newtonian fluids to measure their viscosity using our microcapillary device, namely, water and ethylene glycol at 44% volume concentration. We have used glycerol at this dilution because its density, *ρ*, is then practically identical to ethylene glycol at 44% volume concentration.

## Results

We study fluid flow inside a capillary microchannel using a pressure-driven flow using the experimental set-up described in the Materials section. In this method, a pressure difference is set and the velocity of the fluid-air interface inside the microchannel is measured.

### Experimental results: One fluid system

Our experiments consist of measuring the average position of the front as the fluid fills the capillary.

#### Fluid front velocity is approximately constant

**Fig 3:**
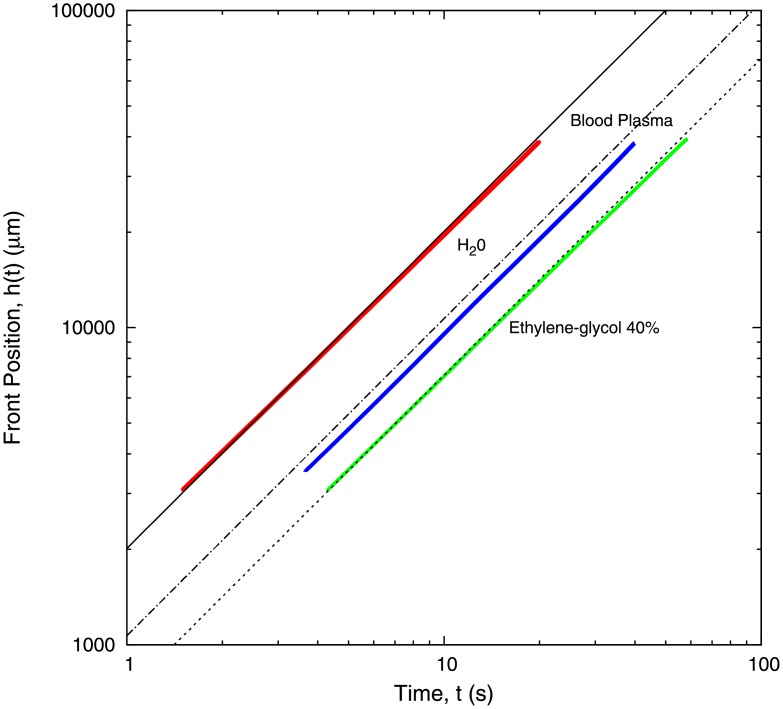
Figure showing the position of the front as a function of time for water, ethylene glycol at 40% dilution, and blood plasma. The value of the exponent *ν* as defined in Eq (1) is *ν* = 0.98 for water, *ν* = 0.98 for ethylene glycol, and *ν* = 1.01 for blood plasma. Experiments were carried out with a liquid column of height *H* = 0.290 m. The value of the height of the capillary is *b* = 300 *μ*m. Thick lines correspond to experimental measures. The dashed line shows theoretical results for water, dotted line corresponds to theoretical results for blood plasma, and narrow-dashed line, to ethylene glycol.

Fig 3 shows the position of the fluid front, *h*(*t*), as a function of time at a column height of *H* = 0.290 *m*. At this pressure the channel is filled in less than 50 seconds. We observe that, for all three liquids, the mean position of the front behaves as

$$\begin{array}{c} h\left(t\right)\approx t^{\nu } \end{array}$$ (1)

where *ν* ≃ 1, consistent with a constant front velocity. Note that this behaviour is different from the predicted by Washburn’s law. According to Washburn’s result, the front velocity should be expected to decay as *t*^−1/2^.

According to Eq (1), the velocity of the front is $\dot{h}\left(t\right)\approx t^{\nu -1}$. Our experimental results show that the velocity of the front remains approximately constant. The variation of velocity in the whole channel (*l*_*c*_ = 4 *cm*) is 8% for water, 11% for ethylene glycol, and 13% for blood plasma (see Fig 3).

In Table 1, we show the front velocity averaged over the length of the capillary for three different liquids, i.e. water, ethylene glycol, and blood plasma, which is then compared to a linear fit of the data shown in Fig 3. The average velocity is measured from the data shown in Fig 3 (i.e. *h*(*t*) vs time). By measuring the position of the front, *h*(*t*), at different times (i.e. as the front progresses along the length of the micro-channel), we can calculate the associated velocity as the time derivative of *h*(*t*). The average velocity is then obtained as the by taking the mean of all the “instantaneous” values over the length of the microchannel.

**Table 1:** Front velocities averaged over the length of the channel. Experiments have been carried out with a liquid column of height *H* = 290 *mm*. The gap of the capillary is *b* = 300 *μ*m.

Fluid	Mean Velocity [*μm*/*s*]	Linear Fit [*μm*/*s*]	Theoretical [*μm*/*s*]
*H*_2_*O*	1926 ± 42	1919.79 ± 0.08	2000 ± 200
Ethylenglycol 40%	672 ± 20	671.56 ± 0.02	800 ± 100
Blood Plasma	959 ± 61	954.8 ± 0.4	1200 ± 200

#### Changes of the front velocity as the height of the column is varied

**Fig 4:**
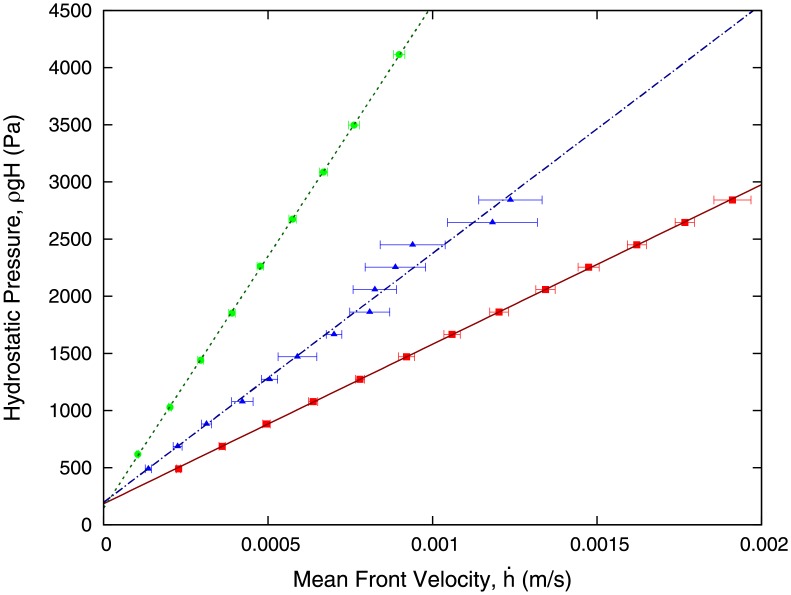
Experimental data of the average velocity of the front for different liquid column heights *H*. These experimental results in addition with the theoretical model Eq (10) is used to estimate the viscosity of each liquid, given in Table 3. Colour code: red corresponds to water, blue to blood plasma, and green to ethylene glycol. The value of the height of the capillary, *b*, is *b* = 300 *μ*m.

We now proceed to measure the velocity of the fluid front as the height of the liquid column, i.e. the pressure, varies. Our results are shown in Fig 4. We observe that our experimental results can be fitted by means of a linear relation:

$$\begin{array}{c} \rho gH=m\dot{h}+n \end{array}$$ (2)

The theoretical model that we present in the Methods section allows us to relate the parameters *m* and *n* to physical properties of the fluid and geometrical parameters of our experimental setup. The quantity *n* corresponds exactly to the capillary pressure associated to the curvature of the front. Regarding the parameter *m*, the theory developed in the Methods section enables us to relate *m* with the viscosity of the liquid. Table 2 shows the values of *m* and *n* obtained form linear fitting of the data shown in Fig 4.

**Table 2:** Fit for the parameters *m* and *n* as defined in Eq (2) from the experimental measurements shown in Fig 4. The gap of the capillary is *b* = 300 *μ*m.

Fluid	m [*Pas*/*μm*]	n [*Pa*]
*H*_2_*O*	1.396 ⋅ 10^6^	184
Ethylenglycol 44%	4.407 ⋅ 10^6^	149
Blood Plasma	2.17 ⋅ 10^6^	201

Within the pressure range in which our experiments take place, the constant velocity regime is preserved until the whole capillary is filled for each value of *H*, which takes an average of *t* = 600 *s*. For each height, *H*, we obtain a velocity profile of the moving fluid front with a dispersion non superior to the 1.82% for water, 1.77% for ethylene glycol at 44% and 2.60% for blood plasma.

### Experimental results: two fluid system

**Fig 5:**
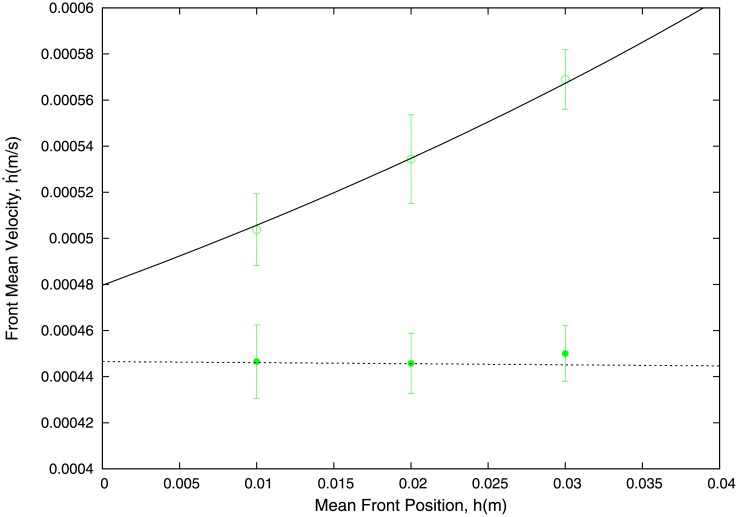
Experimental results showing the increase of the mean front velocity observed in a two-fluid system as the front advances through the microchannel. We are considering a two-fluid system composed by ethylene glycol at 44% pushed by glycerol at 19% (empty circles). We compare this behaviour to that of a single fluid system consisting of ethylene glycol at 44%, where no such acceleration is observed (solid circles). Solid and dashed lines are our theoretical results obtained by using Eq (6) for the two-fluid system (with *η*_1_ < *η*_2_) and the single-fluid system (*η*_1_ = *η*_2_), respectively. The height of the liquid column is *H* = 200 mm and the height of the capillary is *b* = 300 *μ*m.

Our experiments with the two-fluid system consist of measuring the velocity of advancement of the front of the fluid being pushed, which fills the capillary, $\dot{h}$, as a function of its position in the capillary, *h*. In Fig 5 we show experimental results for a two-fluid system consisting of ethylene glycol at 44% pushed by glycerol at 19%. We observe that, as the front of ethylene glycol advances through the microchannel pushed by a liquid column of fixed height, *H* = 200 mm, its velocity increases. By contrast, if the experiment is done with a single-fluid system (ethylene glycol at 44%), we do not observe such an acceleration. Rather, consistent with our results of our one-fluid systems, its front velocity remains constant as it fills the capillary.

This result has non-trivial implications in the way we measure the viscosity of the fluid which fills the capillary. The method developed for measuring the viscosity in a single-fluid system is based on the fact that the advancement of the front occurs at constant velocity. Since this is no longer true for the two-fluid system, we must develop an alternative method.

## Discussion

### Theoretical model

In order to explain the experimental results reported in the Results section, we propose a general mathematical model which accounts for the possibility of dealing with two fluids with positive viscosity contrast.

We assume Darcy’s law with a permeability of a microrectangular cell, $k=\frac{b^{2}}{12}$, where *b* is the height of the micro-channel. We write the velocity of the fluid front inside the microchannel as:

$$\begin{array}{c} \dot{h}\left(t\right)=\frac{b^{2}}{12\eta_{2}}\frac{\Delta P(t)}{h(t)} \end{array}$$ (3)

where *h*(*t*) is the position of the front at time *t* and *η*_2_ is the viscosity to be determined. Δ*P*(*t*) is the total pressure drop:

$$\begin{array}{c} \Delta P\left(t\right)=P_{hyd}-P_{cap}-\Delta P_{R}\left(t\right) \end{array}$$ (4)

where the hydrostatic pressure generated by the column of liquid is *P*_*hyd*_ = *ρgH*, *P*_*cap*_ is the capillary pressure, and, last, the pressure drop generated by the resistance of the microtube connecting the fluid container to the microchannel, Δ*P*_*R*_(*t*), which is now time-dependent, due to the presence of a second fluid of known viscosity, *η*_1_, which progresively fills the microtube:

$$\begin{array}{c} \Delta P_{R}\left(t\right)=\frac{8v_{t}\left(\eta_{1}l_{1}\left(t\right)+\eta_{2}l_{2}\left(t\right)\right)}{r^{2}} \end{array}$$ (5)

where *l*_1_(*t*) and *l*_2_(*t*) are such that *l*_1_(*t*) + *l*_2_(*t*) = *l*_*t*_ at all time, where *l*_*t*_ is the length of the microtube feeding the capillary (see Fig 1). Furthermore, *r* is the radius of the microtube, and *v*_*t*_ is the average flow velocity in the microtube. Both the container and the end of the microchannel are open and exposed to the atmosphere. Other contributions to the pressure drop, e.g. variations of the channel width and microtube cross-section are assumed to be neglegible. To estimate Δ*P*_*R*_(*t*), we have assumed that the microtube connecting the container and the microchannel has a circular cross-section and that the flow inside the microtube obeys Poiseuille’s law.

Taking into account that, due to mass conservation, $\dot{h}bw=v_{t}\pi r^{2}$, we can write the following equation for the velocity of the front inside the microchannel:

$$\begin{array}{c} \dot{h}\left(t\right)=\frac{\rho gH-P_{cap}}{\left(\frac{12\eta_{2}}{b^{2}}+\frac{8b^{2}w^{2}}{\pi^{2}r^{6}}\left(\eta_{1}-\eta_{2}\right)\right)h\left(t\right)+\eta_{2}\frac{8bwl_{t}}{\pi r^{4}}} \end{array}$$ (6)

### One fluid system

The general equation Eq (6) can be used to study our one fluid system by taking *η*_1_ = *η*_2_ ≡ *η*. In this case Eq (6) reduces to:

$$\begin{array}{c} \dot{h}\left(t\right)=\frac{\rho gH-P_{cap}}{\eta \left(\frac{12h(t)}{b^{2}}+\frac{8bwl_{t}}{\pi r^{4}}\right)} \end{array}$$ (7)

### Comparison to experimental results: One fluid system

Eq (7) shows that if our experimental setup is such that the resistance associated to the fluid flow in the microchannel is much smaller than the one associated to the microtube, then we can assume that $\frac{12h(t)}{b^{2}}\ll \frac{8bwl_{t}}{\pi r^{4}}$, which implies that the front velocity is approximately constant:

$$\begin{array}{c} \dot{h}\left(t\right)\approx \frac{\pi r^{4}\left(\rho gH-P_{cap}\right)}{\eta 8bwl_{t}} \end{array}$$ (8)

**Fig 6:**
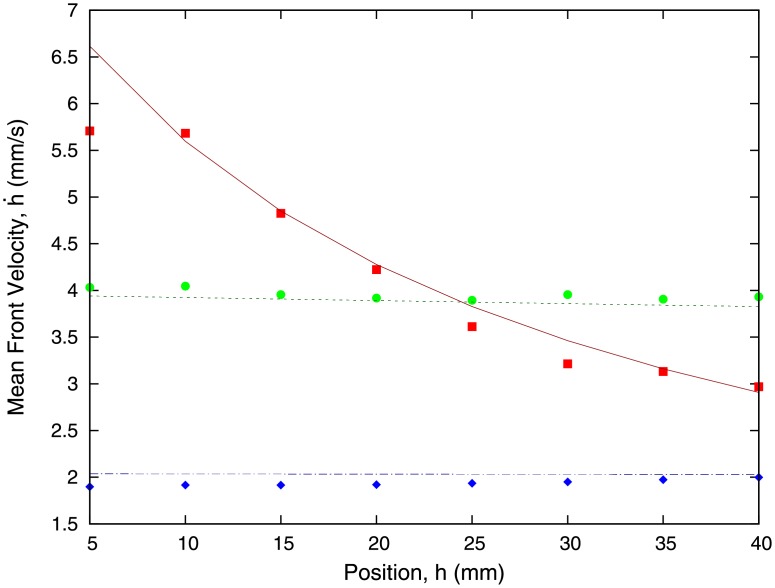
Plot showing the velocity of the front as a function of the position of the front for different heights of the capillary. Experimental results for *b* = 50 *μ*m (red squares), *b* = 150 *μ*m (green dots), and *b* = 300 *μ*m (blue diamonds) are compared to our theoretical model Eq (6). Theoretical results correspond to *b* = 50 *μ*m (red solid line), *b* = 150 *μ*m (green dotted line), and *b* = 300 *μ*m (blue dot-dashed line).

Notice that, as time progresses and *h*(*t*) increases, this approximation becomes less accurate, which implies that this linear regime is valid for either short microchannels or early times. Similarly, the approximation may fail for microchannels with small gap height *b*. This phenomenon is illustrated in Fig 6, where the velocity of the front is shown as a function of the front position. We observe that for larger capillaries (*b* = 150 *μ*m and *b* = 300 *μ*m), the velocity stays approximately constant as the front advances through the capillary, in agreement with our approximation Eq (8). However, as we reduce *b* of the channel, we expect that the approximation leading to Eq (8) fails to hold for smaller capillaries, so that we need to use the full expression for $\dot{h}$, Eq (7). This is confirmed by our experimental results with a capillary of height *b* = 50 *μ*m, where the front velocity is no longer constant as the front advances through the capillary.

**Fig 7:**
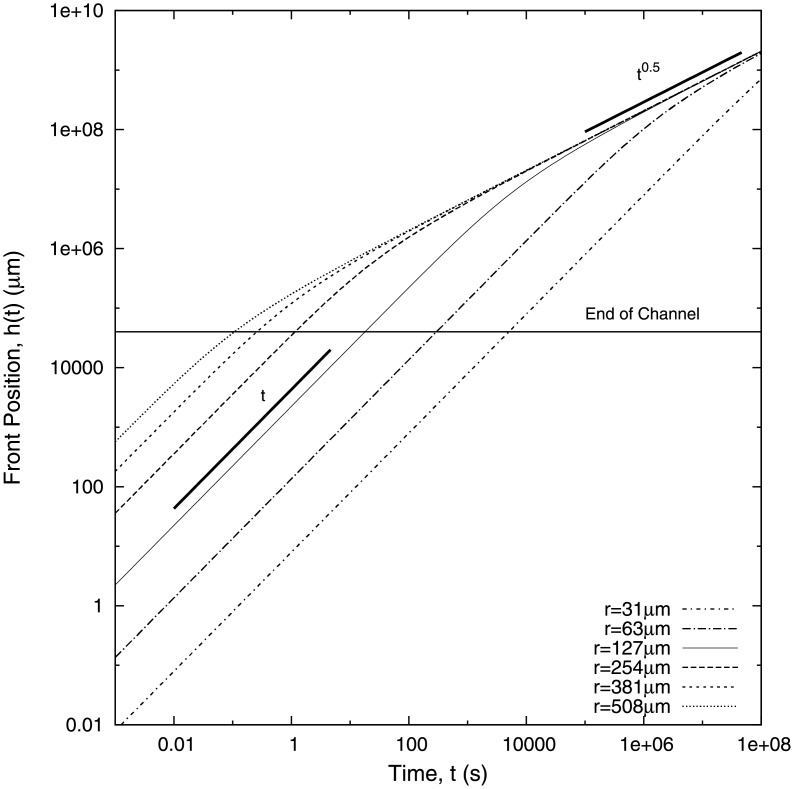
Theoretical front position *h*(*t*) for different values of the radius of the microtube *r*, Eq (9), for water. The value of *r* = 127 *μm* (solid line) corresponds to the radius used in the experimental setup. The thick lines represent *t*^0.5^ and *t* growth exponents. The geometrical parameters used in the plot are those of the experimental setup. The pressure used is *ρgH* − *P*_*cap*_ = 3000*Pa*.

In the general case, when the above assumption does not apply, integration of Eq (7) provides the following expression for the average position of the front, *h*(*t*):

$$\begin{array}{c} \begin{array}{c} h\left(t\right)=-\frac{2}{3}\frac{b^{3}l_{t}w}{\pi r^{4}}+\\ +{\left[{\left(h\left(t_{0}\right)+\frac{2}{3}\frac{b^{3}l_{t}w}{\pi r^{4}}\right)}^{2}+\frac{b^{2}\left(\rho gH-P_{cap}\right)}{6\eta }\left(t-t_{0}\right)\right]}^{\frac{1}{2}} \end{array} \end{array}$$ (9)

from which, in the long time limit, we recover Washburn’s law. In Fig 7 we observe the crossover between both regimes. The early evolution of the front is dominated by a regime where the velocity is approximately constant, whereas for longer times the system decays to the Washburn regime where *h*(*t*) ≈ *t*^1/2^. In particular, in Fig 7, we present numerical results for the crossover between Non-Washburn and Washburn regimes as the radius of the inlet tube is changed. The crossover time is a direct measure of the duration of the linear (Non-Washburn) regime. Our results show that the duration of the linear regime can be controlled by changing the radius: the smaller the radius of the inlet tube, the longer the linear regime.

### Viscosimetry in the one-fluid system

From Eq (8), we can obtain the following relation between the hydrostatic pressure and the (constant) front velocity:

$$\begin{array}{c} \rho gH=\eta \frac{8bwl_{t}}{\pi r^{4}}\dot{h}+P_{cap} \end{array}$$ (10)

By comparing Eqs (2) and (10), we obtain an expression for *m* in terms of the geometrical parameters of our setup and on physical properties of the fluid, in particular its viscosity. Using Eqs (2) and (10), we therefore can measure the viscosity of the fluid:

$$\begin{array}{c} \eta =m\frac{\pi r^{4}}{8bwl_{t}} \end{array}$$ (11)

where *m* is now fitted from experimental data, as shown in Fig 4. The experimental values of the viscosity for several liquids is given in Table 3.

**Table 3:** Table showing our experimental measurements of the viscosity for water, blood plasma, and ethylene glycol. For comparison, we provide estimates for the viscosity of these three liquids found in the literature. Viscosities are given in *mPa* ⋅ *s*.

Fluid	Experimental, *η*_*exp*_	Bibliography
*H*_2_*O*	1.04 ± 0.06	1.002
ethylene glycol at 44%	3.50 ± 0.03	3.4 ± 0.2 [21]
Blood Plasma	1.71 ± 0.7	1.81 [22]

Comparing Eqs (2) and (10), *n* = *P*_*cap*_, so that the capillary pressure associated to the curvature of the front can also be estimated from our experiments. In the case of water, our experimental results show that the capillary pressure is estimated to be *P*_*cap*_ = 184*Pa*. This result can be verified by using the definition of the capillary pressure in terms of the curvature radii of the front:

$$\begin{array}{c} P_{cap}=2\tau \cos\theta \left(\frac{1}{b}+\frac{1}{w}\right) \end{array}$$ (12)

where *τ* is the surface tension, and *θ* is the contact angle of the fluid with the channel wall. By analysing experimental images of the fronts, we estimate the value of the contact angle, *θ* = 108 degree. Then, provided the values of the surface tension for water, *τ* = 0.0728*Nm*^−1^, and those of the parameters *b* = 300 *μ*m and *w* = 1 mm, we estimate the capillary pressure for water to be *P*_*cap*_ = 194.97 Pa. This value is feasible and compatible with our experimental results shown in Table 2.

Our method provides an effective description in which the pressure drop induced by gravity (hydrostatic pressure) is dominant over all other effects, including capillary ones. Our observations (see the example shown in Fig 1) support the assumption that the net effect is a hydrophobic one (i.e. without the liquid column the front recedes). This assumption is further supported by the comparison between Eq (12) and the results obtained from the experimental fit, which we find to agree reasonably well.

#### Effects of the capillary height *b*

Eq (10) can be written in scaling form as:

$$\begin{array}{c} \frac{\pi r^{4}\left(\rho gH-P_{cap}\right)}{8wb^{2}l_{t}}=\eta \frac{\dot{h}}{b} \end{array}$$ (13)

By using the definition of the shear rate, $\dot{\gamma }=\dot{h}/b$, and defining the rescaled pressure, *σ*_*rs*_ as:

$$\begin{array}{c} \sigma_{rs}=\frac{\pi r^{4}\left(\rho gH-P_{cap}\right)}{8wb^{2}l_{t}}, \end{array}$$ (14)

**Fig 8:**
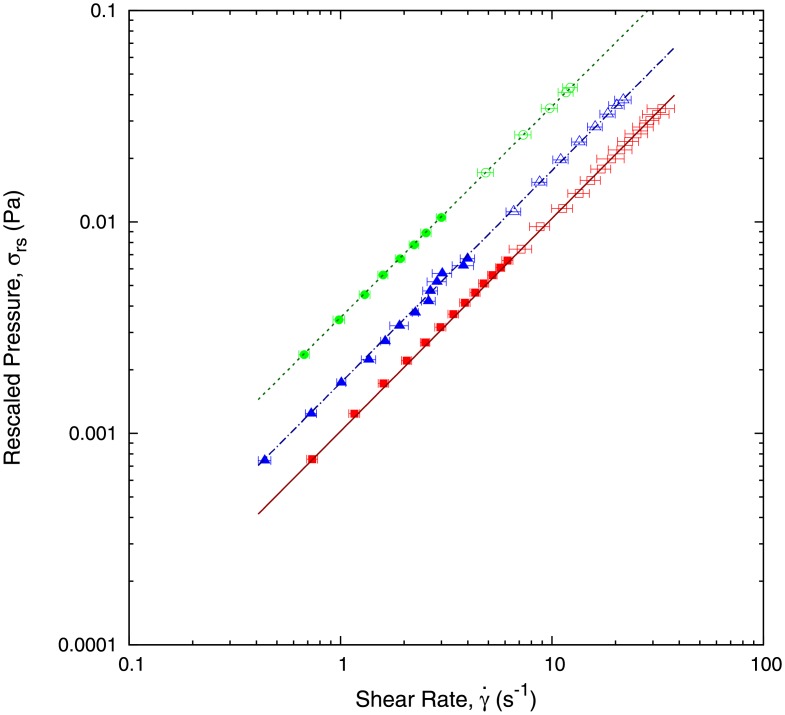
Experimental data represented in terms of the rescaled pressure, *σ*_*rs*_, as defined in Eq (14), and the shear rate, $\dot{\gamma }$. Plotting the experimental results in terms of these variables enables us to directly estimate the viscosity of the fluids (water (red squares), blood plasma (blue triangles), and ethylene glycol at 44% dilution (green circles)) as shown in Eq (15). We have used two capillaries with different heights: *b* = 150 *μ*m (empty symbol) and *b* = 300 *μ*m (solid symbol).

**Fig 9:**
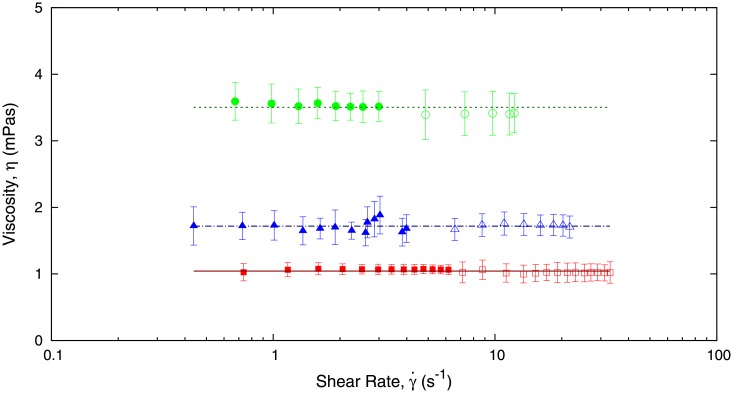
In this plot we use the data shown in Fig 8 to estimate the viscosity of the fluids (water (red squares), blood plasma (blue triangles), and ethylene glycol at 44% dilutions (green circles)) using Eq (15). We observe that for a wide range of values of the shear rate (expanding two orders of magnitude), the viscosity is constant, as it should be expected for a Newtonian fluid. We have used two capillaries with different heights: *b* = 150 *μ*m (empty symbol) and *b* = 300 *μ*m (solid symbol).

Eq (13) can be re-written as:

$$\begin{array}{c} \sigma_{rs}=\eta \dot{\gamma }, \end{array}$$ (15)

independently of geometrical parameters. This scaling form enables us to check that the value of the viscosity in our experiments for the different liquids do not depend on the value of *b*. This is shown in Figs 8 and 9, where we perform experiments with different values of *b*. Additionally, Figs 8 and 9 show that the scaling law Eqs (13)–(15) are satisfied by our experimental results. Furthermore, Fig 9 shows that our methodology can be reliably used as a micro-rheometer.

### Comparison to experimental results: two fluid system

Eq (6) provides a qualitative explanation for the behaviour observed in the experiments reported in Fig 5. If the two-fluid system is such that the fluid that fills the capillary is more viscous than the one which pushes it into the microchannel (i.e. *η*_1_ < *η*_2_), then Eq (6), for a fixed value of *H*, implies that, $\dot{h}$ is an increasing function of *h* (in other words, $\dot{h}$ is an increasing function of time).

The presence of two fluids with different viscosities introduces a term in Eq (6) which is proportional to the viscosity contrast. This term is not present in the single-fluid counterpart of Eq (7). Therefore, applying the same procedure to estimate the unknown viscosity of fluid 2 as we used for the single-fluid case is dubious as the accuracy of the approximation depends on the viscosity contrast which is unknown. We therefore propose a different approach rather than following a the procedure for the one fluid system.

### Viscosimetry of the two fluid system

We now describe a procedure to measure the viscosity of the fluid that fills the capillary, based on the experimental determination of the velocity and position of its front within the microchannel. We start by defining the quantity, *h*_*r*_(*t*), according to:

$$\begin{array}{c} h_{r}\left(t\right)\equiv Ch\left(t\right)+D \end{array}$$ (16)

where *C* is given by:

$$\begin{array}{c} C=1+\frac{1}{E}\left(\frac{\eta_{1}}{\eta_{2}}-1\right), \end{array}$$ (17)

*E* = 3*π*^2^*r*^6^/2*b*^4^*w*^2^ and *D* = 2*b*^3^*wl*_*t*_/3*πr*^4^. Using the definition *h*_*r*_(*t*) and Eq (6), we obtain the following relation:

$$\begin{array}{c} h_{r}\left(t\right)\dot{h}\left(t\right)=\frac{b^{2}}{12\eta_{2}}\left(\rho gH-P_{cap}\right)=a>0 \end{array}$$ (18)

where *a* is a positive constant. Using the fact that $h_{r}\dot{h}\equiv a$ takes a constant value over the length of the capillary, we can derive the value of the unknown viscosity *η*_2_ by measuring the front velocity at two different points of the microchannel. Consider two positions on the microchannel, *h*_1_ and *h*_2_, and the associated (experimentally determined) velocities, ${\dot{h}}_{1}$ and ${\dot{h}}_{2}$. Then $h_{r}\left(h_{1}\right){\dot{h}}_{1}=h_{r}\left(h_{2}\right){\dot{h}}_{2}$, which, by means of the definition of *h*_*r*_ (Eq (16)), we have that:

$$\begin{array}{c} C=\frac{D({\dot{h}}_{2}-{\dot{h}}_{1})}{h_{1}{\dot{h}}_{1}-h_{2}{\dot{h}}_{2}} \end{array}$$ (19)

From Eqs (17) and (19), we can explicitly calculate the value of *η*_2_:

$$\begin{array}{c} \eta_{2}=\frac{\eta_{1}}{1+E\left(\frac{D({\dot{h}}_{2}-{\dot{h}}_{1})}{h_{1}{\dot{h}}_{1}-h_{2}{\dot{h}}_{2}}-1\right)}. \end{array}$$ (20)

Eq (20) provides the value of the unknown viscosity *η*_2_ in terms of the (known) viscosity of the pushing fluid, the geometric characteristics of the experimental setup and experimental measurements of $\dot{h}$ at two different positions along the capillary.

**Fig 10:**
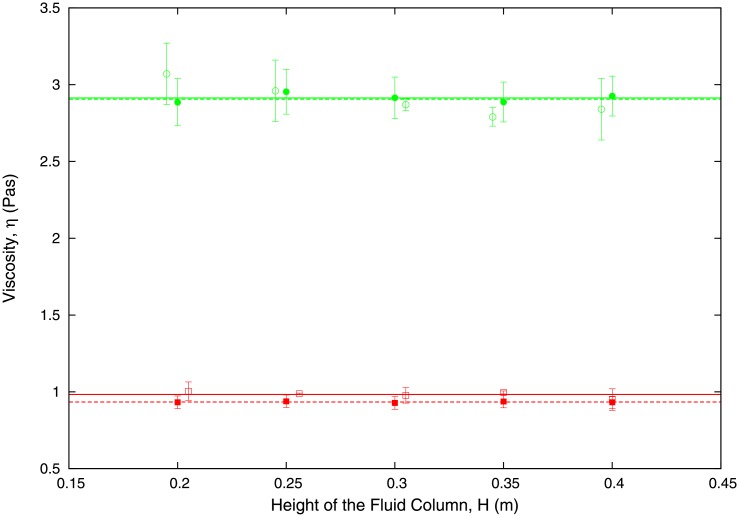
Experimental results for capillary height *b* = 300 *μ*m showing that the value of the viscosity according to our methodology is independent of the height of the liquid column, *H* (see Fig 1). We are considering two-fluid systems composed by water pushed by glycerol at 30% and ethylene glycol at 40% pushed by glycerol at 20%. We compare this behaviour to that of the associated one-fluid system (water and ethylene glycol at 40%). Solid and dashed lines are our theoretical results obtained by using Eq (20) for the two-fluid system (with *η*_1_ < *η*_2_) and the single-fluid system (*η*_1_ = *η*_2_), respectively. The values of the viscosities of water and ethylene glycol measured using the different set-ups (i.e. one- and two-fluids) are given in Table 4. Solid symbols correspond to results obtained with the one fluid system. Empty symbols are associated to measures obtained with the two-fluid set-up.

For this method of measuring the unknown viscosity of the capillary-filling Newtonian liquid to be consistent, the viscosity *η*_2_ should be independent of both the height of the liquid column, *H*, and the height of the capillary, *b*. To check that our method satisfies these consistency criteria, we have performed measurements for different values of *H* and *b*. In order to proceed further, we first fix *b* and we study the behaviour of *η*_2_ as *H* varies. Fig 10 shows the results for the viscosity of water and ethylene glycol for different values of *H*. We further compare the viscosities obtained from the two-fluid model Eq (20) with the estimation obtained with in Eq (11) for benchmark cases (water and ethylene glycol). We observe that, as expected, *η*_2_ is independent of the height of the liquid column, *H*. We also observe an excellent agreement between the estimates produced by our two methods.

In order to check that our method is consistent and therefore produces measurements of *η*_2_ that are independent of *b*, we now proceed to study the behaviour of the viscosity *η*_2_ as *b* varies. Results are shown in Table 4, where we compare the measurements of the viscosities of water and ethylene glycol for two different values of *b*. We also compare the estimates obtained with our two-fluid method to those obtained with the single-fluid method. We observe that both methods consistently predict a value of the viscosity which is independent of *b*. We further show that our estimates of the viscosities for water and ethylene glycol agree with those in the literature.

**Table 4:** Table showing a comparative analysis of the values of the viscosity for water and ethylene glycol obtained by our two different methods for different values of the capillary height. We further compare to viscosity estimates found in the literature (between parentheses) [23, 24]. Slight variations between both sets of values are due to small temperature variations (from 19.3 to 22°C).

	*H*_2_*O* (+Gl30%)		Ethyleneglycol 40% (+GL20%)	
b	One-fluid	Two-fluids	One-fluid	Two-fluids
300	0.93 ± 0.04 (0.94)	0.97 ± 0.03 (0.980)	2.9 ± 0.1 (2.87)	2.9 ± 0.1 (2.87)
200	1.03 ± 0.05 (1.022)	0.97 ± 0.04 (1.022)	3.1 ± 0.2 (2.82)	3.0 ± 0.2 (2.90)

**Fig 11:**
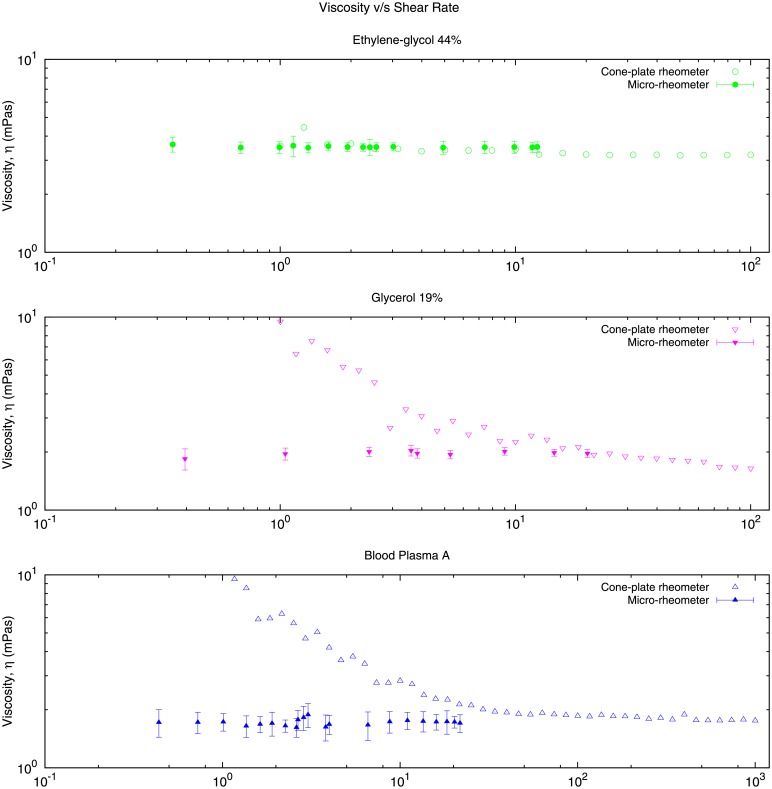
This plot shows experimental values of the viscosity for three Newtonian fluids at low shear rate. We compare the results obtained with our micro-device (solid symbols) to the results produced using a macroscopic commercial cone-plate rheometer (Malvern Kinexus Rotational Rheometer, empty symbols). We observe that the cone-plate rheometer produces spurious results at low shear rates which could be misconstrued as a Non-Newtonian behaviour. By contrast our device is consistent with the Newtonian character of the fluids used in all the range of shear rates.

We have found evidence that our method provides more reliable measures of the viscosity in a wider range of shear rates than a standard, commercial cone-plate rheometre. We present results in Fig 11 in which the viscosities of ethylene glycol 44%, glycerol 19% and blood plasma are measured for different values of the shear rates. We observe that for small shear rates (of the order of 1 s^−1^), the cone-plate rheometre produces spurious results, as the viscosity is not a constant but it depends on the shear rate. This spurious effects are not present in our microfluidic device which produces the right result with a constant viscosity.

## Conclusions

We have proposed an experimental and theoretical framework to study capillary filling at the micro-scale. Our methodology enables us to control the fluid flow regime in such a way as to allows us to characterise the properties of the fluid such as the viscosity. In particular, we have been able to characterise a fluid flow regime where the front position *h*(*t*) ≈ *t*. This regime is a viscous, transient regime which eventually relaxes onto the well-known Washburn regime where *h*(*t*) ≈ *t*^1/2^. Note that this transient regime is different from the inertial regime, which is characterised by much shorter time-scales of the order of 10^−5^ seconds, whereas our linear regime extends to times of the order of minutes. In fact, from the experimental point of view, it is straightforward to control the duration of the linear regime by, for example, changing the geometrical parameters of the device. Furthermore, in addtion to the value of the exponent *ν* to be different from that prescribed by Washburn’s law, the values of the velocity may differ greatly from those expected from Washburn’s prediction, as they depend on the geometrical parameters of the experimental setup. Also, the fact that the velocity of advancement of the front is constant allows for a much simplified method to measure the viscosity and the consequent construction of a viscosimeter.

It is also worth mentioning that our linear regime appears at constant inlet pressure conditions due to the geometry of the experimental set up, rather than by controlling the flow rate and letting the pressure to adapt. We have shown that a possible way to control whether we are within the linear regime is to modify the height of the microchannel, *b*, although other ways are possible such as varying the diameter of the biocompatible microtube connecting the reservoir to the microchannel.

We have further presented an experimental and theoretical methodology that allows us to determine the viscosity of a fluid in a systems of two immiscible liquids by analysing the propagation of the front within a microcapillary. This methodology has the advantage of allowing for the use of very small samples of potentially expensive or rare liquids, as we do not need to fill the device with such liquid. By using such a device and the associated theoretical model, we have been able to explore a non-Washburn regime in which the fluid front within the capillary accelerates or decelerates depending on the viscosity contrast between both liquids: if the fluid that fills the capillary is more viscous than the liquid that pushes it into the capillary, then the front accelerates, and viceversa.

Under these conditions we cannot use the methodology proposed in one-fluid systems to measure viscosities of Newtonian fluids, so we have devised a new method. This new method is based on, for a fixed height of the liquid column, measuring the front velocity at two different positions within the microcapillary. We have checked that the viscosity so obtained does not exhibit spurious dependencies on experimental factors such as the height of the liquid column, *H*, or the value of the capillary gap, *b*. Using this method, we obtain results consistent with those reported in the literature.

The flow regime studied in this paper, in which we can observe both acceleration and decelaration of the fluid front, may induce to misinterpret the data regarding the properties of the fluid flow regime being observed. The theoretical approach that we have devised in this paper allows us to reliably measure the viscosity of Newtonian fluids regardless of the fluid regime introduced by our experimental setup. This theoretical framework is the stepping stone towards studying more complex fluids such as whole blood and other non-Newtonian fluids, as it allows to separate spurious dependencies produced by experimental artifacts from genuine non-Newtonian effects.

This methodology has allowed us to design and calibrate a micro-viscosimeter to determine the viscosity with measurements over a wide range of shear rate, extending about two orders of magnitude. Our device is capable of obtaining the correct value of the viscosity of Newtonian fluids for low values of the shear rate without the need of *ad hoc* corrections, which are necessary when using other types of viscometers.

Finally, we have shown evidence that our method is more reliable than a standard, commercial cone-plate rheometer for small shear rates, as shown in Fig 11. Whereas the cone-plate rheometre produces spurious effects at low shear rates (in this case, predicting that the viscosity of a Newtonian fluid exhibits shear-rate dependence), our microfluidic device avoids these spurios effects and produces the right result with a constant viscosity. This effect is typical and present in several rheometers for fluids with low viscosities measured at low shear rates.

## References

[1] Tabeling P. Introduction to Microfluidics. Oxford University Press; 2005.

[2] Bocquet L, Tabeling P. Physics and technological aspects of nanofluidics. Lab on a Chip. 2014;14:3143 10.1039/C4LC00325J 25046581

[3] Agresti JJ, Antipovc E, Abatea AR, Ahna K, Rowata AC, Barete JC, et al Ultrahigh-throughput screening in drop-based microfluidics for directed evolution. Proc Natl Acad Sci. 2010;107:4004 10.1073/pnas.0910781107 20142500 PMC2840095

[4] Abatea AR, Hung T, Marya P, Agresti JJ, Weitz DA. High-throughput injection with microfluidics using picoinjectors. Proc Natl Acad Sci. 2010;107:19163 10.1073/pnas.1006888107 20962271 PMC2984161

[5] Bezagu M, Errico C, Chaulot-Talmon V, Monti F, Tanter M, Tabeling P, et al High Spatiotemporal Control of Spontaneous Reactions Using Ultrasound-Triggered Composite Droplets. J Am Chem Soc. 2014;136:7205–7208. 10.1021/ja5019354 24785681

[6] Lee W, Aminia H, Stone HA, Carlo DD. Dynamic self-assembly and control of microfluidic particle crystals. Proc Natl Acad Sci. 2010;107:22413 10.1073/pnas.1010297107 21149674 PMC3012521

[7] Atencia J, Beebe DJ. Controlled microfluidic interfaces. Nature. 2005;437:648 10.1038/nature04163 16193039

[8] Ledesma-Aguilar R, Nistal R, Hernandez-Machado A, Pagonabarraga I. Controlled drop emission by wetting properties in driven liquid filaments. Nat Materials. 2011;10:367 10.1038/nmat2998 21478882

[9] Al-Housseini TT, Tsai PA, Stone HA. Control of interfacial instabilities using flow geometries. Nat Physics. 2012;8:747–750. 10.1038/nphys2396

[10] Yu F, Horowitz MA, Quake SR. Microfluidic serial digital to analog pressure converter for arbitrary pressure generation and contamination-free flow control. Lab on a Chip. 2013;13:1911 10.1039/c3lc41394b 23529280

[11] Guillot P, Panizza P, Salmon JB, Joanicot M, Colin A, Bruneau CH, et al Viscosimeter in an microfluidic chip. Langmuir. 2006;22:6438 10.1021/la060131z 16800711

[12] Srivastava N, Davenport RD, Burns MA. Nanoliter viscometer for analyzing blood plasma and other liquid samples. Anal Chem. 2005;77:383 10.1021/ac0494681 15649032

[13] Degre G, Joseph P, Tabeling P, Lerouge S, Cloitre M, Ajdari A. Rheology of complex fluids by particle image velocimetry in microchannels. Appl Phys Lett. 2006;89:024104 10.1063/1.2221501

[14] Quere D. Inertial capillarity. Europhys Lett. 1997;39:533–538. 10.1209/epl/i1997-00389-2

[15] Tas NR, Haneveld J, Jansen HV, Elwenspoek M, van den Berg A. Control of interfacial instabilities using flow geometries. Appl Phys Lett. 2004;85:3274.

[16] Reyssat M, Courbin L, Reayssat E, Stone HA. Imbibition in geometries with axial variation. J Fluid Mech. 2008;615:365 10.1017/S0022112008003996

[17] Queralt-Martin M, Pradas M, Rodriguez-Trujillo R, Arundell M, CorveraPoire E, Hernandez-Machado A. Pinning and avalanches in hydrophobic microchannels. Phys Rev Lett. 2011;106:194501 10.1103/PhysRevLett.106.194501 21668164

[18] Wahsburn EW. The dynamics of capillary flow. Phys Rev. 1921;17:273 10.1103/PhysRev.17.273

[19] McDonald JC, Duffy DC, Anderson JR, Chiu DT, Wu H, Schueller OJA, et al Fabrication of microfluidic systems in poly(dimethylsiloxane). Electrophoresis. 2000;21:27 10.1002/(SICI)1522-2683(20000101)21:1<27::AID-ELPS27>3.0.CO;2-C 10634468

[20] Rodriguez-Villarreal I. Microfluidic device for blood plasma separation and magnetic cell manipulation. PhD Thesis. Universitat de Barcelona; 2011.

[21] Global M. Ethyleneglycol product guide. ME Global; 2008.

[22] Baskurt OK, Hardemanand MR, Rampling MW, Meiselman HJ. Handbook of hemorheology and hemodynamics. IOS Press; 2007.

[23] Cheng NS. Formula for viscosity of glycerol-water mixture. Ind Eng Chem Res. 2008;47:3285–3288. 10.1021/ie071349z

[24] The Engineering Toolbox;. Available from: http://www.engineeringtoolbox.com/ethylene-glycol-d_146.html.

